# The Chemokine MIP-1α/CCL3 impairs mouse hippocampal synaptic transmission, plasticity and memory

**DOI:** 10.1038/srep15862

**Published:** 2015-10-29

**Authors:** Elodie Marciniak, Emilie Faivre, Patrick Dutar, Claire Alves Pires, Dominique Demeyer, Raphaëlle Caillierez, Charlotte Laloux, Luc Buée, David Blum, Sandrine Humez

**Affiliations:** 1 Université de Lille, F-59000 Lille, France; 2 Inserm UMR_S1172, Jean-Pierre Aubert Research Centre, F-59000 Lille France; 3 CHU-Lille, F-59000 Lille France; 4 Centre de Psychiatrie et Neurosciences, UMR_S 894, Faculté de Médecine, Université Paris Descartes, 75014, Paris; 5 SFR DN2M, 59000 Lille France

## Abstract

Chemokines are signaling molecules playing an important role in immune regulations. They are also thought to regulate brain development, neurogenesis and neuroendocrine functions. While chemokine upsurge has been associated with conditions characterized with cognitive impairments, their ability to modulate synaptic plasticity remains ill-defined. In the present study, we specifically evaluated the effects of MIP1-α/CCL3 towards hippocampal synaptic transmission, plasticity and spatial memory. We found that CCL3 (50 ng/ml) significantly reduced basal synaptic transmission at the Schaffer collateral-CA1 synapse without affecting NMDAR-mediated field potentials. This effect was ascribed to post-synaptic regulations, as CCL3 did not impact paired-pulse facilitation. While CCL3 did not modulate long-term depression (LTD), it significantly impaired long-term potentiation (LTP), an effect abolished by Maraviroc, a CCR5 specific antagonist. In addition, sub-chronic intracerebroventricular (icv) injections of CCL3 also impair LTP. In accordance with these electrophysiological findings, we demonstrated that the icv injection of CCL3 in mouse significantly impaired spatial memory abilities and long-term memory measured using the two-step Y-maze and passive avoidance tasks. These effects of CCL3 on memory were inhibited by Maraviroc. Altogether, these data suggest that the chemokine CCL3 is an hippocampal neuromodulator able to regulate synaptic plasticity mechanisms involved in learning and memory functions.

The immune system plays a central role in controlling neuronal plasticity and memory. Most of the knowledge concerning the relationships between the immune system and learning and memory processes focused on the role of soluble inflammatory cytokines, particularly IL-1β, IL-6 and TNF-α^1^,^2^. IL-1β was notably described as a double edge sword finely modulating synaptic processes and memory abilities in physiological conditions^3^,^4^ but playing a detrimental role in chronic delivery conditions^5^,^6^,^7^.

Besides cytokines, another class of inflammatory factors, namely chemokines, has been associated to neuroinflammatory and neurodegenerative conditions^8^,^9^,^10^. So far, about 50 chemokines binding to 20 chemokine receptors, having various physiological and pathological properties, have been discovered. Chemokines contribute to numerous aspects of immune function as leukocyte trafficking from circulation to tissue, immune surveillance and inflammatory processes^11^,^12^. Chemokines have also been suggested to exhibit important roles in the central nervous system where they regulate brain development, neurogenesis, neuroinflammation and neuroendocrine functions^13^,^14^.

Recently, chemokines emerged as potential modulators of neuronal activity^15^ but, so far, only few have been described to regulate neuronal activity and plasticity. It is now well established that SDF-1/CXCL12 and MCP-1/CCL2 modulate electrical activity of dopaminergic and dorsal root ganglia neurons^15^,^16^. CCL2 enhances neuronal excitability and synaptic transmission in hippocampal slices^17^ while fraktalkine/CX_3_CL1 impairs LTP in the CA1 region and the dentate gyrus^18^,^19^. However, the role of most chemokines regarding hippocampal-dependent plasticity and spatial memory remains ill-defined^20^.

MIP-1α/CCL3 is classically described as a neutrophil chemoattractant. This chemokine has been found in the central nervous system and its cognate receptors, CCR1 and CCR5, have been reported to be expressed by astrocytes, microglia and neurons^21^,^22^,^23^,^24^,^25^. CCL3 is thought to play a role in neurodegenerative conditions. It has been particularly found associated with temporal lobe epilepsy^22^,^26^,^27^,^28^ and Alzheimer’s disease (AD)^9^,^10^. CCL3 levels in the serum of AD patients have been associated with mood disturbances in cross-sectional analysis^29^. CCL3 has been reported expressed by neurons and microglia in post-mortem brains from AD patients^30^ and upregulated in experimental models mimicking both amyloid and Tau deposits^31^,^32^,^33^. In addition, a CCL3 polymophism has been associated with AD^34^,^35^ and CCL3 secretion was found dependent on apoplipoprotein ε4, the greatest genetic risk factor for sporadic AD^36^. However, even if previous works showed that CCL3 may affect neuronal Ca^2+^ signaling^25^ and neuronal network activity *in vitro*^37^, whether CCL3 modulates hippocampal synaptic plasticity and memory remains unknown so far.

In the present study, we evaluated effects of CCL3 towards hippocampal synaptic transmission, plasticity and spatial memory. We found that CCL3 significantly reduced basal synaptic transmission and LTP at the Schaffer collateral-CA1 hippocampal synapses and impaired spatial and long-term memory as seen using the Y-maze and passive avoidance tasks. These detrimental effects were found dependent on the CCR5 receptor. CCL3 is thus prone to regulate synaptic plasticity mechanisms involved in learning and memory processes.

## Results

### *Ex-vivo* effects of CCL3 on hippocampal basal synaptic transmission and short-term plasticity

**Figure 1:**
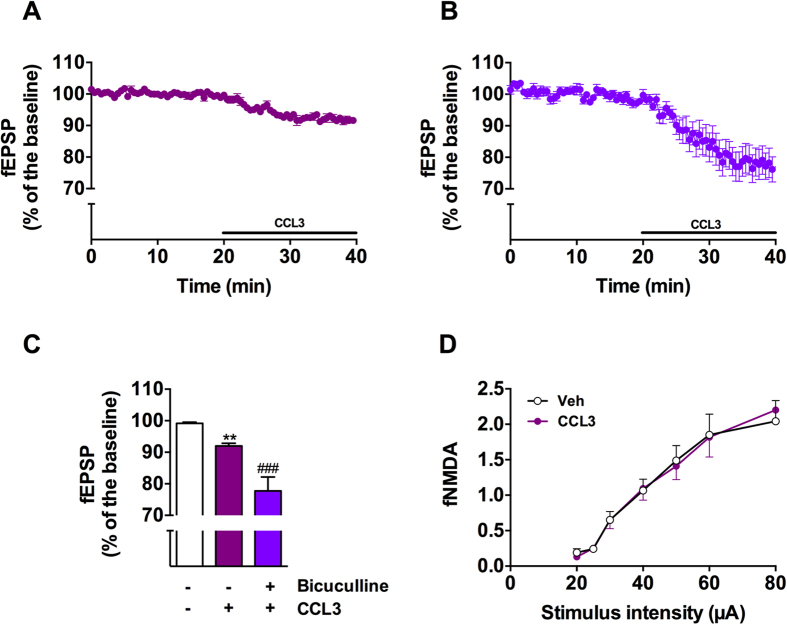
CCL3 decreases basal synaptic transmission without impacting NMDA receptors. (**A**) Effects of CCL3 (50 ng/mL) on the fEPSPs amplitude (n = 12). (**B**) Effect of CCL3 on the fEPSPs amplitude in the presence of bicuculline (10 μM; n = 11). Note that in our experiments, bicuculline (10 μM) caused a fEPSP increase reaching 10.0 ± 1.5% of the baseline (n = 4). To compare the effect of CCL3 on the basal synaptic transmission with or without bicuculline, an I/V curve was realized before baseline recording to adjust the intensity of the stimulus of each experiment to elicit a fEPSP of 35% of the maximum. This intensity was kept constant throughout the CCL3 application. Each point represents mean ± SEM normalized to the baseline values preceding the application of CCL3. Horizontal bar represents chemokine application (50 ng/mL, 20 min). (**C**) Representative histograms of fEPSP variation during the last five minutes of drug application. **p < 0.01 vs. baseline; ###p < 0.001 vs. CCL3 alone, using One-way ANOVA and post-hoc LSD Fisher test. (**D**) CCL3 (50 ng/mL) does not affect NMDAR-mediated field potential input-output curves.

Stable field excitatory potentials (fEPSPs) were elicited in the CA1 stratum radiatum of mouse hippocampal slices by electrical stimulation of the Schaffer collateral (0.033 Hz). Bath application of CCL3 (50 ng/mL) for 20 min caused a significant reduction of fEPSPs to 92 ± 0.8% of baseline (Fig. 1A,C; CCL3: p = 0.0078 vs. Baseline, using One-Way ANOVA followed by LSD Fisher test, n = 12) which was maximal after 10 min of chemokine application and remained stable (Fig. 1A). Noteworthy, effect of CCL3 was reversible upon washout of the drug, supporting that fEPSP reduction was not due to slice run down (not shown). In the presence of bicuculline (10 μM), a GABA_A_ receptor antagonist, CCL3-induced fEPSPs drop was significantly greater (Fig. 1B,C), reaching 77.8 ± 4.4% of the baseline (CCL3+Bic: p < 0.0001 vs. CCL3, using One-Way ANOVA followed by LSD Fisher test, n = 11). This result indicated that CCL3-induced decreased of synaptic transmission could be partially masked by a small decrease of GABAergic transmission. We also examined the effect of CCL3 upon the NMDA component previously described to be modulated by inflamatory mediators^38^. To this aim, we have recorded NMDA fields (fNMDA) on slices perfused in a low (0.1 mM) Mg^2+^ aCSF supplemented with the AMPA/kainate receptor antagonist NBQX (10 μM). As depicted on Fig. 1D, CCL3 did not modify the amplitude of the fNMDA (p > 0.05, using Two-Way ANOVA) supporting that CCL3-induced decrease of hippocampal fEPSPs is not related to the NMDA-mediated response but to an impaired AMPA-dependent synaptic transmission.

**Figure 2:**
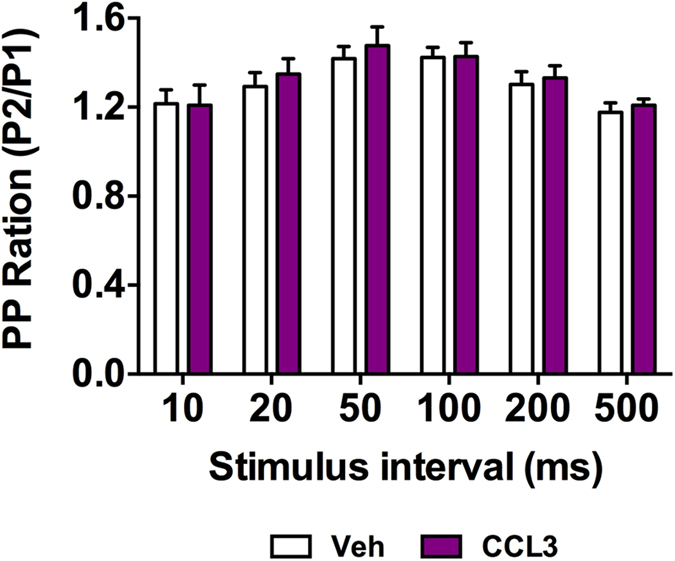
CCL3 does not affect paired-pulse facilitation. Paired-pulse ratio remains unchanged in the presence of CCL3 at the different stimulus interval studied (10, 20, 50, 100, 200 and 500 ms, n = 5).

Paired-pulse facilitation (PPF) represents a form of presynaptic short-term plasticity, whose variation is generally associated to transmitter release probability. PPF was induced by applying stimuli separated by different time intervals (10, 20, 50, 100, 200 and 500 ms). We found no effect of CCL3 at any interval (p > 0.05; Fig. 2) supporting the idea that CCL3 does not mediate its effects through the modulation of neurotransmitter release from presynatic terminals. Together, our data support that CCL3 effects on basal synaptic transmission is ascribed to an AMPA-dependent post-synaptic effect.

### *Ex-vivo* CCL3 effects on long-term synaptic plasticity

**Figure 3:**
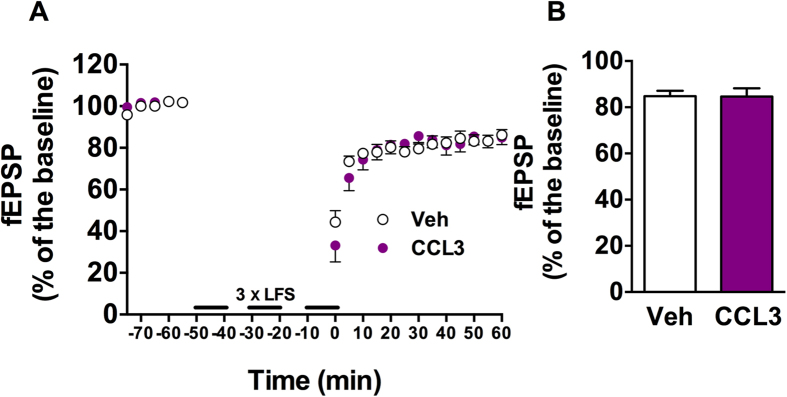
CCL3 does not impact long-term depression. (**A**) LTD was evoked using LFS consisting of three train of 1500 pulses at 2 Hz delivered every 10-min between each train in normal condition or in the presence of CCL3 (50 ng/mL). In order to normalized the pre-LTP or pre-LTD signal between different slices, an I/V curve was realized before baseline recording to adjust the intensity of the stimulus of each experiment to elicit a fEPSP of 35% of the maximum. This intensity was kept constant throughout the CCL3 application. Each point represents the average of 3 responses (mean ± SEM) evoked every 30 s in seven different slices. (**B**) Effect of CCL3 on fEPSP after 60 min post LFS (n = 7 for each condition).

**Figure 4:**
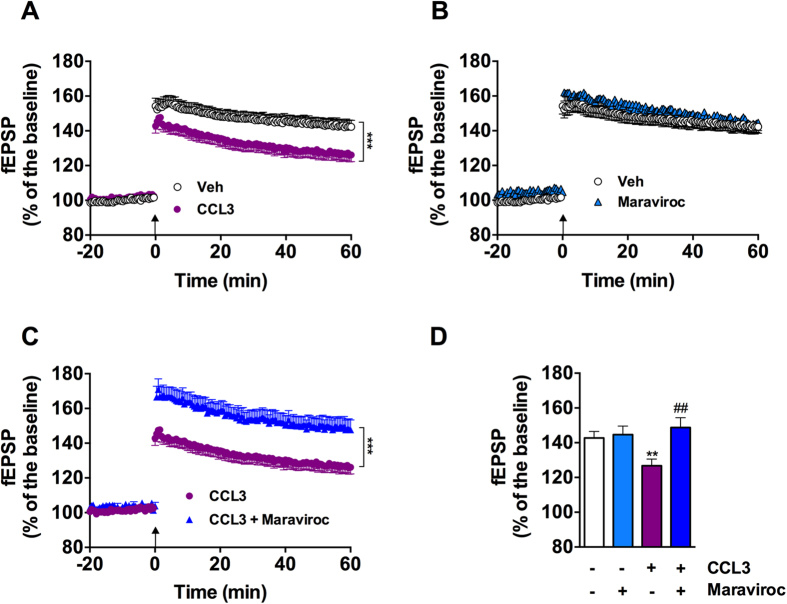
CCL3 decreases hippocampal long-term potentiation expression via an effect on CCR5 receptors. (**A**) CCL3 treatment (50 ng/mL) induces a significant decrease of the LTP induced by 4 trains at high frequency, symbolized by the arrow (control vehicle condition, n = 31; CCL3 treatment, n = 16; ***p < 0.001 vs. Vehicle, Two-way ANOVA). (**B**) Maraviroc (15 nM), a CCR5 antagonist does not affect LTP (control vehicle condition, n = 31; maraviroc, n = 11; p > 0.05 vs. Vehicle, Two-Way ANOVA). (**C**) Co-treatment with CCL3 (50 ng/mL) and Maraviroc (15 nM) prevents CCL3 effects on LTP expression (n = 16, ***p < 0.001 vs. CCL3 alone, Two-way ANOVA). In order to normalized the pre-LTP or pre-LTD signal between different conditions, an I/V curve was realized before baseline recording to adjust the intensity of the stimulus of each experiment to elicit a fEPSP of 35% of the maximum. This intensity was kept constant throughout the CCL3 application. (**D**) Representative histograms of fEPSP variation during the last five minutes in the different experimental conditions (**p < 0.01 vs. Vehicle, ##p < 0.01 vs. CCL3 alone using One-Way ANOVA followed by a LSD Fisher post-hoc test).

We next evaluated CCL3 effects upon long-term synaptic plasticity, namely long-term depression (CA1-LTD) and potentiation (CA1-LTP). LFS elicited a robust LTD that was similar regardless treatment (Vehicle: 84.8 ± 2.3% vs. CCL3 50 ng/mL: 84.6 ± 3.6% n = 7/group; p = 0.96 using Student’s t-test; Fig. 3B). In sharp contrast, LTP was significantly affected by the chemokine (Vehicle vs. CCL3: p < 0.001 using Two-way ANOVA, Fig. 4A; Vehicle: 142.7 ± 3.7% vs. CCL3: 126.7 ± 3.8%, p < 0.01; n = 31 and 16 respectively, using One-Way ANOVA followed by LSD Fisher post-hoc test, Fig. 4D). CCL3-induced LTP impairment was totally prevented by Maraviroc (15 nM), a competitive antagonist of CCR5 receptor (CCL3 vs. CCL3+Maraviroc: p < 0.001, Two-way ANOVA, Fig. 4C; CCL3: 126.7 ± 3.8% vs. CCL3+Maraviroc: 144.6 ± 4.9% ; p = 0.002; n = 16/group, Fig. 4D). Maraviroc alone did not impact LTP (Control vs. Maraviroc: p > 0.05, Two-way ANOVA, Fig. 4B; Control: 142.7 ± 3.7% vs. Maraviroc: 148.7 ± 5.6%, p > 0.05; n = 31 and 11 respectively, Fig. 4D). Together, these data support that CCL3 exerts a detrimental effect on long-term potentiation through activation of the CCR5 chemokine receptor.

### *In vivo* effects of CCL3 on hippocampal synaptic activity

**Figure 5:**
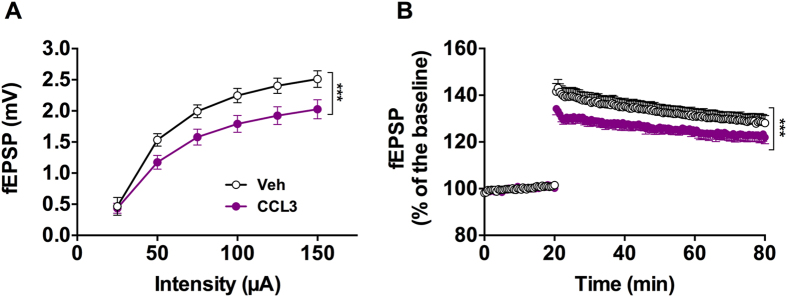
Intracerebroventricular administration of CCL3 induces impairments in hippocampal synaptic transmission and plasticity. (**A**) Sub-chronic i.c.v administration of CCL3 impairs hippocampal input-output curve (n = 5–6/group, ***p < 0.001 vs. Vehicle-injected mice, TWO-Way ANOVA). **(B)** Sub-chronic i.c.v administration of CCL3 induces a significant decrease of the LTP after the induction by 4 trains at high frequency (n = 5–6/group, ***p < 0.001 vs. Vehicle-injected mice, TWO-Way ANOVA). In order to normalize the pre-LTP signal between different conditions, an I/V curve was realized before baseline recording to adjust the intensity of the stimulus of each experiment to elicit a fEPSP of 35% of the maximum. This intensity was kept constant throughout the protocol.

To address CCL3 effects *in vivo* upon synaptic transmission and plasticity, animals were intracerebroventricularly injected with CCL3 during 7 days. Intracerebroventricular (i.c.v) injections of CCL3 induced a significant decrease of the hippocampal basal synaptic transmission as observed at the level of the input-output curve (***p < 0.001 using Two-way ANOVA, Fig. 5A; n = 5–6 animals/group). Further, these sub-chronic injection of CCL3 impaired LTP. Indeed, CCL3 treated mice exhibiting a significantly smaller LTP than Vehicule-treated animals (***p < 0.001, Two-way ANOVA, Fig. 5B; n = 5–6 animals/group).

### CCL3 effect on memory

**Figure 6:**
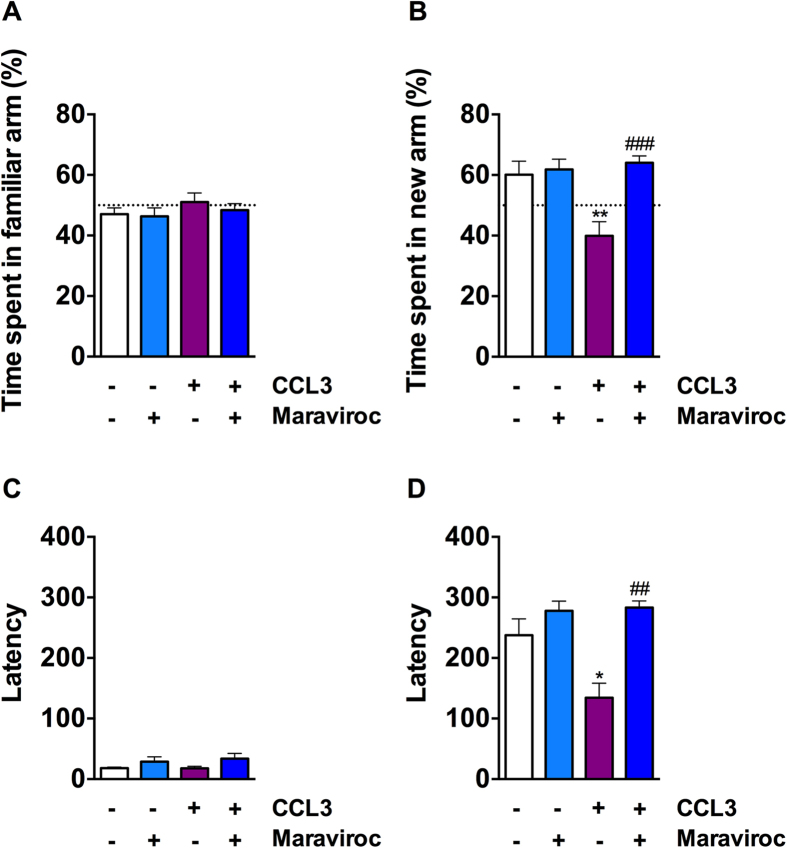
Intracerebroventricular injection of CCL3 impairs memory via an effect on CCR5 receptors. (**A**,**B**) Y-maze task. (**A**) During the acquisition phase, mice from all groups explored the maze equally, spending similar amount of time in each available arm (start and other/familiar; P > 0.05, One-way ANOVA, n = 6–10 per group). (**B**) Following 20 min of retention (test phase), preference for the novel arm was abolished in the CCL3 injected-group. (Veh vs. CCL3: **p < 0.01; One-way ANOVA followed by a post-hoc LSD Fisher test, n = 6–10 per group). This impairment was alleviated by the CCR5 antagonist Maraviroc, co-injected with CCL3 (CCL3 vs. CCL3+Maraviroc: ###p < 0.001; One-way ANOVA followed by a post-hoc LSD Fisher test, n = 6–10 per group) while the antagonist alone did not exert an effect by itself. **(C**,**D**) Passive avoidance task. (**C**) During the training phase, there was no difference between experimental groups in the latency to enter the dark compartment (p > 0.05, One-way ANOVA, n = 6–10 per group). (**D**) As expected, following 24 h of retention, the latency to enter in the dark substantially increased in Veh-treated mice supportive of preserved memory. Latency to enter the dark compartment was significantly reduced in CCL3-injected mice (Veh vs. CCL3: *p < 0.05; One-way ANOVA followed by a post-hoc LSD Fisher test, n = 6–10 per group), an effect prevented by the CCR5 antagonist Maraviroc (CCL3 vs. CCL3+Maraviroc: ##p < 0.01; One-way ANOVA followed by a post-hoc LSD Fisher test, n = 6–10 per group). The antagonist alone did not exert an effect by itself.

In order to evaluate the impact of CCL3 upon memory, we finally evaluated the effect of i.c.v injections of CCL3 on spatial memory abilities using the spatial version of the Y-maze task, a short term memory test as well as passive avoidance, a long-term memory paradigm. Dependency on CCR5 receptor was evaluated following co-administration of the antagonist Maraviroc with CCL3. During the exposure phase of the Y-maze task, all experimental groups (injected with Vehicle and CCL3 +/−Maraviroc; n = 6–10/group) explored the maze equally, spending a similar amount of time in the two available arms (P > 0.05; Fig. 6A. Figure 6 represents the percentage spent in the familial arm which is equal to 50%; significance vs. 50%: p > 0.05 using One-sample t-test). Velocity was similar for all groups (P > 0.05, not shown). During the test phase, we found that preference for the novel arm was significantly reduced in CCL3-injected mice (Veh: 60.1 ± 4.5% vs. CCL3: 39.9 ± 4.6%; P < 0.01 using One-Way ANOVA followed by LSD Fisher post-hoc test, Fig. 6B). Interestingly, effect of CCL3 was reversed following co-injection of the CCR5 antagonist Maraviroc (CCL3: 39.9 ± 4.6% vs. CCL3+Maraviroc: 64.0 ± 2.3% vs; P < 0.001 using One-Way ANOVA followed by LSD Fisher post-hoc test, Fig. 6B). Maraviroc alone did not change impact of short-term memory (Veh: 60.1 ± 4.5% vs. Maraviroc: 61.9 ± 3.4%; P > 0.05 using One-Way ANOVA followed by LSD Fisher post-hoc test, Fig. 6B). Sub-chronic intracerebroventricular administration of CCL3 was also found to impair long-term memory as shown using the passive avoidance paradigm. During the training phase, there was no difference in the latency to enter in dark compartment regardless treatment (p > 0.05 using One-way ANOVA followed by a post-hoc LSD Fisher test; Fig. 6C). Following 24 h of retention, in the memory test phase, the latency to reach the dark compartment was significantly reduced in CCL3-treated animals compared to Veh group (Veh: 237 ± 27s vs. CCL3: 134 ± 24s; P < 0.05, using One-way ANOVA followed by a post-hoc LSD Fisher test; Fig. 6D). This supported that the subchronic delivery of the chemokine impairs long-term memory. Detrimental effect of CCL3 was reversed following co-injection of the CCR5 antagonist Maraviroc (CCL3: 134 ± 24s vs. CCL3+Maraviroc: 283 ± 11s; P < 0.01 using One-Way ANOVA followed by LSD Fisher post-hoc test; Fig. 6D). Maraviroc alone did not change impact of short-term memory (Veh: 237 ± 27 s vs. Maraviroc: 278 ± 16s; P > 0.05 using One-Way ANOVA followed by LSD Fisher post-hoc test, Fig. 6D).

## Discussion

In addition to their role in the immune system, chemokines have been suggested to play important roles in the central nervous system during brain development, neurogenesis or neuroinflammation^13^,^14^. Some of them such as CCL2 and fraktalkine have been described as modulators of hippocampal neuronal activity^14^,^15^,^20^, but the impact of most members of this class of molecules upon hippocampal synaptic plasticity and spatial memory remains unknown. To our knowledge, the present study is the first describing the detrimental effects of CCL3 towards hippocampal synaptic transmission, plasticity and memory.

Our data support that CCL3 impairs basal synaptic transmission *ex-vivo* and *in vivo*. While other inflammatory mediators as IL-6 have been described to modulate synaptic transmission through an enhancement of both AMPA- and NMDA-mediated synaptic responses^38^, our data support that CCL3 impairements of synaptic transmission were mostly ascribed to change in AMPA activity as our data indicate that CCL3 was unable to modify fMNDA recorded in the presence of the AMPAR blocker NBQX, in low Mg^2+^ concentration conditions. The effect of CCL3 towards basal synaptic transmission remained, and was found to be significantly greater, in presence of bicuculline. This observation supported that in addition to acting on basal AMPA-dependent transmission, CCL3 may also slightly reduce GABAergic transmission. Such effect has been previously described for another member of the chemokine family, namely CXCL14, shown to inhibit GABA-mediated postsynaptic currents and the GABAergic tonic currents^39^. Nevertheless, a specific study of the effect of CCL3 on GABAergic interneurons will be needed to highlight a possible depressive effect of CCL3 on GABAergic neurons. Some chemokines, as CXCL8, SDF-1/CXCL12 or CCL2, have also been suggested to control neurotransmitter release^16^,^40^,^41^,^42^. Our PPF data however indicated that the CCL3-dependent reduction of basal synaptic transmission induced by CCL3 was rather post-synaptic.

To get further insights on the effects of CCL3 towards synaptic plasticity in the CA1 area of hippocampus, we have evaluated the impact of the chemokine towards LTP and LTD, two forms of long-term plasticity considered to be the cellular correlates of memory^43^,^44^. Interestingly, our data demonstrated that CCL3 significantly impaired LTP, while sparing LTD. In addition, as CCL3 do not modify NMDA field potentials, we can suggest that CCL3-induced LTP impairment is NMDA-independant. Importantly, our *in vivo* data demonstrate that detrimental effects of CCL3 upon synaptic transmission and plasticity were also observed in hippocampal slices from animals sub-chronically injected with CCL3 in ventricles. The detrimental effect of CCL3 appears mediated by CCR5 receptor, as it was reversed by the specific antagonist Maraviroc. CCR5 is one of the cognate CCL3 receptor previously reported to be expressed by astrocytes, microglia and neurons^23^,^24^,^25^,^45^,^46^. Our data extend the known neuromodulatory role of the CCR5 receptor. Indeed, CCR5 was found to enhance pain perception at inflammatory sites and to modulate glutamate release in human neocortex^47^,^48^. In hippocampus, the two chemokines known to activate CCR5 receptors, i.e. CCL3 and CCL5, have been found to regulate calcium signalling and to inhibit the frequency of spontaneous glutamatergic excitatory postsynaptic currents^25^. Interestingly, the CCR5 antagonist itself did not impact LTP *per se*. While we cannot rule out that the amount of endogeneous CCL3 present or released in hippocampal slices is too low, our observations suggest that the tonic activity of the receptor is not involved in plasticity as for instance reported for IL-1β^3^,^4^.

Detrimental effect of CCL3 on hippocampal LTP both *ex-vivo* and *in vivo* is in accordance with impaired memory abilities measured using the two-step Y-maze and passive avoidance tasks, following daily i.c.v. injections of the chemokine. While Y-maze evaluates short-term memory, it is not surprising that chronic hippocampal impairments promoted *in vivo* by sub-chronic icv CCL3 injections leads to hippocampal dysfunction reflected by Y-maze alterations. Similar changes have indeed been found in several conditions associated to synaptic plasticity alterations^49^,^50^. However, our data also point that i.c.v CCL3 injections leads to impaired score using the passive avoidance task, a long-term memory paradigm previously linked to LTP^51^,^52^ and described to be impaired when LTP is affected^53^. Other data previously reported that intra-nasal administration of CCL3 impairs social recognition^54^. CCL3 may thus be able to exert detrimental effect on several forms of cognitive behaviours. While other mechanisms could be involved, notably the ability of CCL3 to favour glial activation and the surge of other cytokines, our data support that effects of CCL3 towards memory is related to its ability to impair synaptic plasticity. This view is in line with the attenuation of Aβ-induced memory alterations previously reported in CCR5 knock-out transgenic mice^55^. It also raises the more general view that CCL3 upsurge, as seen in various pathological conditions such as temporal lobe epilepsy or AD, associated with major cognitive dysfunctions^9^,^10^,^21^,^26^,^27^,^28^ might mediate detrimental synaptic impairments.

CCR5-deficient mice were previously shown to demonstrate memory alterations in link with astrocyte activation and Aβ deposits^56^. However, other studies demonstrate that CCR5 knock-out only prevent mice from Aβ or in HIVgp120-induced-cognitive deficits, while the sole deletion of CCR5 did not exert effect upon memory by itself 55,57. In line with the latter studies, blockade of CCR5 by Maraviroc impacts plasticity and memory only in the presence of CCL3 without any effect by itself, finally suggesting that the tonic CCR5 activity is not necessary to memory formation in non-pathological conditions. Altogether, our data highlight for the first time that abnormal activation of the CCR5 pathway is prone to impair synaptic processes leading to memory loss, supporting a role in pathologies associated with CCL3 upsurge.

Underlying cellular and molecular mechanisms associated to the detrimental effect of CCR5 activation by CCL3 towards synaptic transmission, plasticity and memory remain unclear. Basal synaptic transmission and plasticity are known to be dependent on AMPA receptor activity and trafficking^43^. Passive avoidance has also been associated to an increase of *in vivo* field potential and to LTP and, in this behavioural paradigm, performances have been linked to the synaptic delivery of AMPARs in CA1 neurons^51^,^52^. Alterations of synaptic plasticity and memory induced by CCL3 through the activation of CCR5 seen in our paradigms are possibly related to AMPA receptor regulation and trafficking changes at the post synaptic membrane. Nervertheless, it has been also shown that CCR5 is also expressed by glial cells^21^,^22^,^23^,^24^,^25^ and it is now well known that astrocytes contribute to the regulation of synaptic activity, plasticity and memory^58^,^59^,^60^. Therefore, we cannot exclude that modifications of astrocyte functions (reuptake of neurotransmitter, liberation of gliotransmitter) operated by CCL3 could also participate to its detrimental effects upon synaptic activity and memory. Further studies will be necessary to decipher the precise mechanisms linking CCL3/CCR5 axis to synaptic functioning and memory emergence.

In conclusion, our study highlights a new neuromodulatory function for CCL3 in the hippocampus, bringing new insights on the potential role exerted by this chemokine in pathologies associated with memory dysfunctions. Whether it may represent a valuable therapeutic target will deserve future studies.

## Materials and Methods

### Drugs

The following drugs were used in the present study: Recombinant CCL3-Macrophage Inflammatory Protein 1 alpha (MIP-1α) (Peprotech Inc. Rocky Hill, NJ, USA), the CCR5 antagonist Maraviroc (Tocris Bioscience, Bristol, UK), the GABA_A_ receptor antagonist, Bicuculline (Sigma Aldrich, Saint Quentin Fallavier, France), the AMPA receptor antagonist 2,3-dioxo-6-nitro-1,2,3,4-tetrahydrobenzoquinoxaline-7-sulfonamide (NBQX) (Tocris Bioscience, Bristol, UK). All agents were diluted directly in the aCSF from stock solutions prepared in distilled water or in dimethylsulfoxide.

### Animals

Experiments were performed using 8–12 weeks-old C57BL/6 male mice (Charles River, France). All animals were maintained in standard animal cages under conventional laboratory conditions (12 h/12 h light/dark cycle, 22 °C), with *ad libitum* access to food and water. The animals were maintained in compliance with European standards for the care and use of laboratory animals and experimental protocols approved by the local Animal Ethical Committee (n°CEEA342012 on December 12, 2012 from CEEA Nord-Pas-de-Calais),

### Hippocampal slices preparation

Mice were killed by cervical dislocation. Whole brains were rapidly removed from the skull and immersed for 1 min in ice-cold artificial cerebrospinal fluid (ACSF) solution containing (in mM): NaCl 117, KCl 4.7, CaCl2 2.5, MgCl2 1.2, NaH2PO4 1.2, NaHCO3 25 and glucose 10. The ACSF was continuously oxygenated with 95% O2, 5% CO2 to maintain the proper pH (7.4). The hippocampi were removed in ice-cold ACSF and then transverse 400 μm thick slices were performed at 4 °C with a chopper (McIlwain Tissue Chopper, TC752). Slices were then placed in a holding chamber of ACSF, oxygenated and kept at room temperature for at least 1 h before recording.

### Electrophysiological recordings

Individual slices were transferred to a submersion-type slice-recording chamber (BSC1, Scientific System Design Inc. Little Ferry, NJ USA). Slices were maintained at 31–32 °C and constantly superfused at the rate of 2.5 ml/min with ACSF, continuously oxygenated with 95% O2, 5% CO2. Field excitatory postsynaptic potentials (fEPSPs), mostly resulting from the activation of AMPA receptors, were evoked by electrical stimulation of Schaffer collaterals afferent to CA1 and commissural fibers in the stratum radiatum and record using an ACSF-filled glass micropipette (2–5 MΩ). Stimulations were delivered using a concentric bipolar stimulated electrode (25 μm exposed tip, FHC Inc, Bawdoin ME, USA) and consist in 100 μs constant current square pulses, applied at 0.033 Hz. Evoked responses were amplified (DAM 80, World Precision Instruments, Sarasota FL USA) digitized with a M serie PCI board (National Instrument, Nanterre FRANCE) and monitored/analyzed on-line using Win LTP software (Anderson and Collingridge software). To analyze synaptic transmission efficiency, input/output plots were constructed individually for each slice by applying single stimulus in increments of 25 μA from 0 to 200 μA. The intensity of the stimulus was adjusted in each experiment to elicit a fEPSP of 35% of the maximum and was kept constant throughout the experiment. In all experiments baseline synaptic transmission was monitored for 20 min before drug administration or delivering repetitive stimulations. When experiments were conducted in presence of bicuculline (10 μM), we separated CA3 and CA1 by a knife cut to prevent the propagation of epileptiform discharges. In our experiments, bicuculline alone (10 μM) caused a fEPSP increase reaching 10.0 ± 1.5% of the baseline (n = 4). Further, to compare the effect of CCL3 on the basal synaptic transmission with or without bicuculline, an I/V curve was realized before baseline recording to adjust the intensity of the stimulus of each experiment to elicit a fEPSP of 35% of the maximum. To evaluate short-term synaptic interaction, we delivered paired-pulse stimuli with interstimulus interval from 10 to 500 ms and each paired-pulse was delivered with a 120 sec interval and measured paired-pulse facilitation (PPF). Long-term potentiation (LTP) was evoked by high frequency stimulations (HFS) using four trains of 100 pulses at 100 Hz delivered every 10s and fEPSP were recorded during 60 min after HFS. Long-term depression (LTD) was evoked using low frequency stimulations (LFS) consisting of three train of 1500 pulses at 2 Hz (0.2 ms pulse-width) delivered every 10-min between each train. For these experiments the synaptic strength before and after LFS was measured every 5 min using the mean of EPSP evoked with three single stimuli (0.1 ms pulse-width; 10 s interval). Any change in synaptic strength was expressed relative to the control level (100%) and the mean responses during the last 5 minutes were expressed as a percentage of the control.

NMDAR-mediated fEPSPs were recorded in slice perfused with low-Mg^2+^ (0.1 mM) ACSF supplemented with the AMPA/Kainate receptor antagonist 2,3-dioxo-6-nitro-1,2,3,4-tetrahydrobenzoquinoxaline-7-sulfonamide (NBQX, 10 μM). CCL3 impact on NMDAR-mediated fEPSPs was investigated by an evaluation of the input/output curves. Curves were constructed to assess the responsiveness of NMDA glutamate receptor subtype-dependent responses to electrical stimulation. The amplitude of three averages fEPSPs was plotted as a function of stimulation intensity (20 to 100 μA).

### Surgical procedures and injections

Bilateral canulae (C235G-3.0/SPC with a removable dummy wire; Plastics One) were stereotaxically implanted into lateral ventricule (coordinates with respect to bregma: −0.7 mm anteroposterior [AP], +/−1.5 mm mediolateral [ML], -2 mm dorsoventral [DV], according to the Paxinos and Franklin mouse brain atlas (Paxinos and Watson, 2005) in anesthetized mice (1.5% isoflurane). Animals were allowed to recover for 1 week. The following 15 days, animals were habituated to the contention and injection procedure. Animals were then injected with either CCL3 and Vehicule for electrophysiological experiments or with CCL3, Maraviroc, CCL3+Maraviroc or Vehicle (PBS) for behavioural evaluations. Injections were performed in awake and freely moving mice. Animals were injected with 1 microliter (per ventricle) of a solution containing either Vehicle (PBS, pH 7.5), CCL3 (250 ng/μL in PBS), Maraviroc (500 ng/μL) or Maraviroc + CCL3 at the rate of 0.4 μl/min via cannulae PE50 tubing (Plastics One) connected to a 10 μL Hamilton syringe pump system (KDS310; KD Scientific). The tubing was left in place for another 1 min at the end of each injection, and the cannulae capped to prevent reflux of the injected solution. In a first group of animals, LTP was assessed after the 7^th^ injection. Memory was assessed in another group of animals following 7 (Y-maze) or 9 (Passive avoidance) injections (see below). Placement of canula were confirmed following post-mortem histology (not shown).

### Y-maze test

Short-term spatial memory was assessed in a spontaneous novelty-based spatial preference Y-maze test as described after 7 daily injections (the last injection occur between the exposure phase and the test phase)^33^. Each arm of the Y-maze was 28 cm long, 6.2 cm wide, with 15-cm-high opaque walls. Different extramaze cues were placed on the surrounding walls. Sawdust was placed in the maze during the experiments and mixed between each phase. Allocation of arms was counterbalanced within each group. During the exposure phase, mice were placed at the end of the ‘start’ arm and were allowed to explore the ‘start’ arm and the ‘other (familiar)’ arm for 10 min (beginning from the time the mouse first left the start arm). Access to the third arm of the maze (‘novel’ arm) was blocked by an opaque door. The mouse was then removed from the maze and returned to its home cage for 20 min. In the test phase the mouse was placed again in the ‘start’ arm of the maze, the door of the ‘novel’ arm was removed and the mouse was allowed to explore the maze for 5 min (from the time the mouse first left the start arm). The amount of time the mouse spent in each arm of the maze was recorded during both exposure and test phases using EthovisionXT (Noldus Information Technology, Wageningen, The Netherlands). For the exposure phase, we calculated the percentage of time spent in the ‘other’ (familiar) arm vs. the ‘start’ arm.

### Passive avoidance conditioning

Mice which were subjected to Y maze was then used in passive avoidance paradigm. The appartus is a two-chambered box consisting of a lighted safe chamber and a dark shock chamber separated by a trap door. Briefly, mice were trained 30 min after the eighth CCL3 injection. During training, mice were placed in the safe chamber of the bock. After the opening of the trap door mice entered into the dark box at will. We recorded latency to enter the dark chamber where a slight footshoock (0.5 mA, 1s) was delivered. Memory was tested following a 24 hour retention and 30 min after the ninth daily injection. Mice were replaced in the chamber and latency to enter in the dark chamber was measured.

### Sacrifice of the animals and tissue post-processing

Animals were sacrificed 30 minutes following the last behavioural analysis (i.e. following 9 daily bilateral intracerebroventricular injections). Briefly, mice were transcardiacally injected with cold NaCl 0.9%. Hemispheres were separated. The left hemispheres were post-fixed in 4% of paraformaldehyde for 5 days, then deshydrated in a PBS sucrose solution at 30% and cut using a cryostat. Hippocampi from the right hemispheres were dissected out for biochemical experiments. For the latter, tissue was homogeneized in RIPA buffer (25 mM Tris-HCl, 150 mM NaCl, 1% NP40, 1% sodium deoxycholate, 0.1% SDS, pH 7.6).

### ELISA

For ELISA experiments, homogenates were processed as follows. After sonication and homogeneisation for 1 h in RIPA bufer, samples were centrifugated at 12000 g for 15 min at 4 °C and supernatants were collected. CCL3 was measured in homogenates (300 μg of protein in 50 μL) using ELISA following manufacturer’s instructions (R&D system). We found that a 9-daily bilateral intracerebroventricular injection of CCL3 achieve hippocampal levels of CCL3 reaching 138 ± 35 pg/mL as compared to PBS-injected controls.

### Statistics

Results are expressed as mean ± SEM. Statistics were performed using either Student’s t-test as well as One or Two-way analysis of variance (ANOVA), followed by a post-hoc Fisher’s LSD test using Graphpad Prism Software. P values  < 0.05 were considered significant.

## Additional Information

**How to cite this article**: Marciniak, E. *et al.* The Chemokine MIP-1α/CCL3 impairs mouse hippocampal synaptic transmission, plasticity and memory. *Sci. Rep.* **5**, 15862; doi: 10.1038/srep15862 (2015).
